# Curcumin Improves Epithelial Barrier Integrity of Caco-2 Monolayers by Inhibiting Endoplasmic Reticulum Stress and Subsequent Apoptosis

**DOI:** 10.1155/2021/5570796

**Published:** 2021-10-06

**Authors:** Xinxin Zhou, Mengting Ren, Jinpu Yang, Hanghai Pan, Mosang Yu, Feng Ji

**Affiliations:** ^1^Department of Gastroenterology, The First Affiliated Hospital, Zhejiang University School of Medicine, Hangzhou, Zhejiang Province, China; ^2^Department of Gastroenterology, Zhejiang Provincial People's Hospital, People's Hospital of Hangzhou Medical College, Hangzhou, Zhejiang Province, China

## Abstract

Curcumin is a natural polyphenol and is supposed to possess antioxidant, anti-inflammatory, anticancer, and antiapoptotic properties. Although some studies have reported the therapeutic effects of curcumin on ulcerative colitis (UC), the specific mechanism remains unclear. An in vitro coculture model of Caco-2 and differentiated THP-1 cells was established. After administration of curcumin (10 *μ*M), Western blot analysis was performed to evaluate the protein levels of tight junction (TJ) proteins zonula occludens- (ZO-) 1 and claudin-1. Annexin V-APC/7-AAD assays and flow cytometry were conducted to assess Caco-2 cell apoptosis. The expression levels of oxidative stress and endoplasmic reticulum stress- (ERS-) related molecules were determined by Western blot analysis. Curcumin administration significantly upregulated ZO-1 and claudin-1 protein levels and reduced Caco-2 cell apoptosis. The protein levels of oxidative stress markers inducible nitric oxide synthase (iNOS) and *γ*H2AX and ERS-induced apoptosis-related molecules C/EBP homologous protein (CHOP) and cleaved caspase-12 were significantly downregulated upon curcumin treatment. Furthermore, curcumin administration greatly blocked the protein kinase-like endoplasmic reticulum kinase- (PERK-) eukaryotic translation initiation factor 2*α*- (eIF2*α*-) activating transcription factor 4- (ATF4-) CHOP signaling pathway. Curcumin enhanced intestinal epithelial barrier integrity in the in vitro coculture model by upregulating TJ protein expressions and reducing intestinal epithelial cell apoptosis. The potential mechanisms may be suppression of ERS and subsequent apoptosis.

## 1. Introduction

Ulcerative colitis (UC), one of the major subtypes of inflammatory bowel disease (IBD), is a chronic nonspecific inflammatory disease that mostly affects the distal colon and rectum. Currently, the incidence and prevalence of UC are increasing globally, especially in developing countries [1]. Typical symptoms of UC include bloody diarrhea, abdominal pain, fecal urgency, and weight loss [2]. Patients with UC frequently suffer relapse and are prone to develop UC-associated carcinogenesis [3]. At present, clinical therapies for UC mainly include 5-aminosalicylates (5-ASA), thiopurines, immunosuppressive agents, glucocorticoids, and biological agents [2, 4]. However, long-term usage of these drugs is not effective and often causes severe adverse effects, imposing a heavy burden on society and families.

Although a complex interplay of genetics, immunity, environmental factors, and gut microflora is suggested to contribute to the pathogenesis of UC, its exact etiology and pathogenesis remain elusive [2]. Recently, a series of studies revealed that impairment of intestinal barrier function is one of the major characteristics of UC, and damage to the intestinal epithelium plays a crucial role in the pathogenesis of UC [5–8]. The intestinal epithelial barrier is a physical and biochemical barrier which prevents harmful antigens and pathogens in the gut from accessing the lamina propria and even systemic circulation [8, 9]. It is not surprising that disturbance of gut barrier function can aggravate inflammation and result in the occurrence and progression of UC [6]. However, there is currently no FDA-approved therapy targeting the epithelial barrier [8]. Thus, finding effective drugs that strengthen the intestinal epithelial barrier is critical for the treatment of UC.

The endoplasmic reticulum (ER) is a major eukaryotic organelle that modulates various physiological processes including protein processing, calcium storage, lipid biosynthesis, and drug detoxification [10]. As an essential component of the intestinal barrier, intestinal epithelial cells (IECs) process strong ER function for biosynthesis of large amounts of protein [11]. Disturbances of many homeostatic processes such as nutrient deprivation, energy deficiency, changes in calcium homeostasis, lipid overload, ischemia, and hypoxia lead to accumulation of unfolded or misfolded proteins in the ER, triggering endoplasmic reticulum stress (ERS) [10, 11]. Severe or persistent ERS may impair the homeostasis and function of IECs and even lead to apoptosis [12], suggesting the potential role of ERS in maintaining intestinal epithelial barrier function.

The pathogenesis of UC is closely related to excessive ERS. Elevated expression levels of ERS-associated molecules were observed in the intestinal epithelium of patients with active UC [13–15]. ERS within IECs plays a key role in aggravating intestinal barrier damage and inflammation [13, 16, 17]. Therefore, targeting ERS-related signaling in IECs is a potential therapy for UC.

Curcumin, a natural polyphenolic compound separated from turmeric, is supposed to possess antioxidant, anti-inflammatory, anticancer, and antiapoptotic properties [18, 19]. The agent has been widely studied in various diseases because it is inexpensive and has very low toxicity [20]. Recently, several studies demonstrated that curcumin could attenuate cell apoptosis and tissue damage caused by excessive ERS [21, 22]. Animal experiments revealed that curcumin played an anti-inflammatory role in murine experimental colitis [23–26]. Although some studies have reported that curcumin exerts therapeutic effects on UC, the specific mechanism remains unclear [27–29].

In the present study, we found that curcumin enhanced intestinal epithelial barrier integrity in the coculture model by upregulating tight junction (TJ) protein expressions and reducing intestinal epithelial cell apoptosis via suppression of ERS. Specifically, the PERK-eIF2*α*-ATF4-CHOP signaling pathway and the cleavage of caspase-12 were significantly inhibited.

## 2. Materials and Methods

### 2.1. Chemicals and Kits

Curcumin was obtained from Selleck Chemicals (Houston, TX, USA) and dissolved in dimethyl sulfoxide (DMSO) to obtain an initial concentration of 10 mM and stored at −80°C. Phorbol-12-myristate-13-acetate (PMA) and lipopolysaccharide (LPS) were bought from Sigma-Aldrich (St. Louis, MO, USA). An Annexin V-APC/7-AAD kit was bought from Keygen Biotechnology (Nanjing, China). Rabbit antibodies against zonula occludens- (ZO-) 1 (8193), claudin-1 (13255), *γ*H2AX (Ser139; 9718), glucose-regulated protein 78 (GRP78; 3183), protein kinase-like endoplasmic reticulum kinase (PERK; 5683), eukaryotic translation initiation factor 2*α* (eIF2*α*; 5324), p-eIF2*α* (3398), activating transcription factor 4 (ATF4; 11815), and glyceraldehyde-3-phosphate dehydrogenase (GAPDH; 2118) were purchased from Cell Signaling Technology (Danvers, MA, USA). Rabbit antibodies inducible nitric oxide synthase (iNOS; ab178945), C/EBP homologous protein (CHOP; ab179823), and caspase-12 (ab62484) were obtained from Abcam (Cambridge, UK). HRP-labelled goat anti-rabbit secondary antibody (HA1001) was purchased from HuaBio (Hangzhou, China).

### 2.2. Cell Culture and Treatment

The Caco-2 and THP-1 cell lines were purchased from the American Type Culture Collection (ATCC; Manassas, VA, USA) and were grown in DMEM medium (Gibco, Carlsbad, CA, USA) and RPMI 1640 medium (Gibco), respectively, supplemented with 10% fetal bovine serum (FBS; Gibco) at 37°C with 5% CO_2_. Caco-2 cell passage was performed at a ratio of 1 : 2 or 1 : 3 using 0.25% trypsin-ethylene diamine tetraacetic acid (EDTA) solution (Gibco) every 2–3 days. Human colon cancer cell line Caco-2 has been demonstrated to form epithelial monolayers in vitro closely resembling the physiological intestinal epithelium. Human monocyte cell line THP-1 can be differentiated into macrophages with PMA. Similar to other studies [30–32], we established a coculture model of Caco-2 cells and activated THP-1 cells as described in our published article [33]. Briefly, Caco-2 cells (2 × 10^5^ cells/well) were seeded in 6-well culture inserts (Corning Costar, NY, USA) and cultured for 18 days. Culture medium was changed every one to two days. THP-1 cells (1.5 × 10^6^ cells/well) were seeded in 6-well plates and treated with PMA (100 ng/mL) for 48 h to differentiate into macrophages. Next, the Transwell insert (Caco-2) was placed into the culture well (THP-1), and LPS (10 ng/ml) was added to the lower chamber. The two cell lines were cocultured for 24 h. Once the coculture model was established, for the curcumin group, curcumin was added to the upper chamber of the coculture model to achieve a final concentration of 10 *μ*M for 48 h. And the control group received an equal volume of cell culture medium with 0.1% DMSO.

### 2.3. Flow Cytometry Detection of Cell Apoptosis

Cell apoptosis was assessed using an Annexin V-APC/7-AAD cell apoptosis detection kit and flow cytometry. After 48 h of curcumin (10 *μ*M) treatment, Caco-2 cells were collected by 0.25% trypsin without EDTA, washed twice with phosphate-buffered saline (PBS), and resuspended in 500 *μ*L of 1x binding buffer. A total of 5 *μ*L of Annexin V-APC staining solution and 5 *μ*L of 7-AAD staining solution were added to the cell suspension, and the mixture was incubated at room temperature for 5 min in the dark before being analyzed by a CytoFLEX flow cytometer (Beckman Coulter, Fullerton, CA, USA). The cells were considered apoptotic when they were either APC+/7-AAD− (early apoptotic) or APC+/7-AAD+ (late apoptotic).

### 2.4. Quantitative Real-Time Polymerase Chain Reaction (qRT-PCR)

After curcumin treatment (10 *μ*M) for 48 h, total RNA of Caco-2 cells was isolated using the TRIzol reagent (Takara Biotechnology, Shiga, Japan), and reverse transcription was performed using a PrimeScript™ RT Master Mix kit (Takara Biotechnology) according to the manufacturer's protocol. Afterwards, qRT-PCR was performed using a SYBR Green Premix Ex Taq kit (Takara Biotechnology) and carried out in the ABI 7500 real-time system (Thermo Fisher Scientific, Waltham, MA, USA). The primers were synthesized by Sangon Biotech (Shanghai, China), and the sequences are listed in Table 1.

### 2.5. Western Blot Analysis

After curcumin administration (10 *μ*M) for 48 h, total protein of Caco-2 cells was extracted with cell lysis buffer (Cell Signaling Technology), and protein quantification was performed using the BCA assay (Thermo Fisher Scientific) according to the manufacturer's instructions. Protein (40 *μ*g) was loaded on gels for sodium dodecyl sulfate-polyacrylamide gel electrophoresis (SDS-PAGE) and then transferred onto polyvinylidene difluoride (PVDF) membranes. After blocking with 5% bovine serum albumin (BSA) for 2 h at room temperature, the desired primary antibody was added and placed at 4°C overnight. The membranes were incubated with HRP-labelled goat anti-rabbit secondary antibody on the following day. Finally, the membranes were soaked with enhanced chemiluminescence (ECL) reagents (Millipore, Bedford, MA, USA) and visualized using the chemiluminescence imaging system (Clinx Scientific Instruments, Shanghai, China) with exposure time ranging from 20 s to 1 min. Protein bands were quantified by densitometry using ImageJ software (NIH, Bethesda, MD, USA), which attributed pixel values to each band, and densitometry was analyzed for three independent experiments. Densitometry units of the p-eIF2*α* protein bands were normalized to the corresponding bands for eIF2*α*, and the other bands were normalized to GAPDH as the loading control in the same lane.

### 2.6. Statistical Analysis

Experimental results are expressed as the mean ± standard deviation (SD). Statistical analyses were performed using two-sided unpaired Student's *t*-test by GraphPad Prism (version 7.0). All experiments were repeated at least three times, and samples were analyzed in triplicate. *P* values < 0.05 were considered significant.

## 3. Results

### 3.1. Effect of Curcumin on Tight Junctions in Caco-2 Monolayers

**Figure 1:**
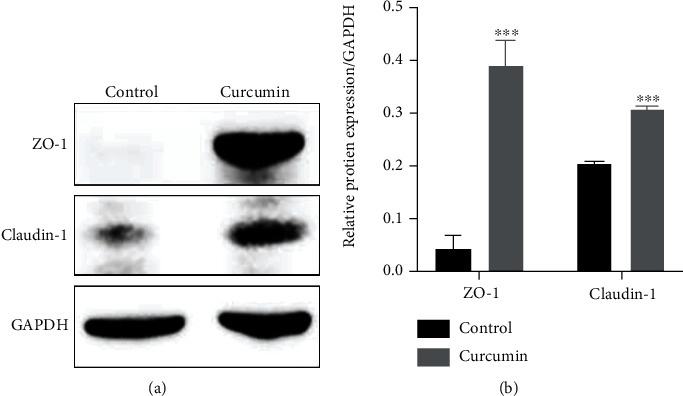
Curcumin treatment enhances tight junction proteins in Caco-2 monolayers. (a) The expression of TJ-related proteins ZO-1 and claudin-1 was determined by Western blot after curcumin administration (10 *μ*M) for 48 h. (b) Protein bands of ZO-1 and claudin-1 were quantified by densitometry, and relative protein expression levels were normalized to GAPDH. Data are shown as the mean ± SD. ^∗∗∗^*P* < 0.001. ZO-1: zonula occludens-1; TJ: tight junction.

Tight junctions are integral components of the intestinal barrier, and dysregulation of TJs contributes to defects of epithelial barrier function [34]. The Caco-2 cell monolayer has been extensively used to mimic the intestinal epithelial barrier. To evaluate Caco-2 monolayer integrity, we detected the protein levels of TJ-related proteins ZO-1 and claudin-1 by Western blot. Our results showed that curcumin treatment (10 *μ*M) for 48 h significantly upregulated ZO-1 and claudin-1 protein levels in Caco-2 cells (Figure 1, *P* < 0.001). In brief, the above results suggest that curcumin enhances the integrity of Caco-2 monolayers by upregulating TJ protein levels.

### 3.2. Effect of Curcumin on Cell Apoptosis of Caco-2 Monolayers

**Figure 2:**
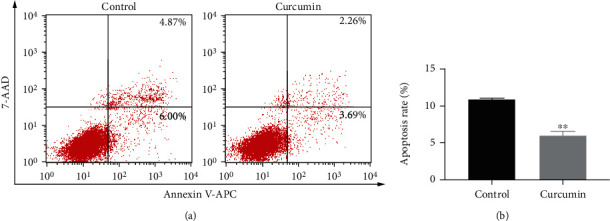
Curcumin inhibits cell apoptosis of Caco-2 monolayers. (a) After curcumin administration (10 *μ*M) for 48 h, cell apoptosis was measured using Annexin V-APC/7-AAD double staining and flow cytometry. (b) Percentage of apoptotic cells. Apoptosis rate was early apoptosis percentage plus late apoptosis percentage. Data are shown as the mean ± SD. ^∗∗^*P* < 0.01.

To investigate the impact of curcumin on cell apoptosis of Caco-2 monolayers, Annexin V-APC/7-AAD assay and flow cytometry were performed. As shown in Figure 2, the mean Caco-2 cell apoptotic rate of the control group was 10.87% (apoptotic rate = early apoptotic rate + late apoptotic rate, mean early apoptotic rate = 6.00%, and mean late apoptotic rate = 4.87%). After curcumin administration (10 *μ*M), the mean apoptotic rate was significantly reduced to 5.95% (mean early apoptotic rate = 3.69% and mean late apoptotic rate = 2.26%; *P* < 0.01), indicating that curcumin exerts a protective effect against apoptosis of Caco-2 cells in the coculture model.

### 3.3. Effect of Curcumin on Oxidative Stress in Caco-2 Monolayers

**Figure 3:**
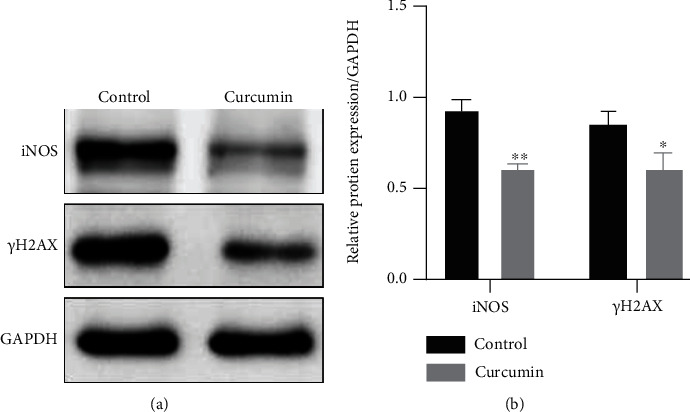
Curcumin suppresses oxidative stress in Caco-2 monolayers. (a) The protein levels of iNOS and *γ*H2AX were detected by Western blot after curcumin treatment (10 *μ*M) for 48 h. (b) Protein bands of iNOS and *γ*H2AX were quantified by densitometry, and relative protein levels were normalized to GAPDH. Data are shown as the mean ± SD. ^∗^*P* < 0.05 and^∗∗^*P* < 0.01. iNOS: inducible nitric oxide synthase.

iNOS and *γ*H2AX are markers for oxidative stress and DNA damage, respectively [35]. In the present study, we found that curcumin administration (10 *μ*M) for 48 h significantly downregulated the protein levels of iNOS and *γ*H2AX in Caco-2 cells compared with those in the control group (Figure 3, *P* < 0.01 and *P* < 0.05), suggesting that curcumin can inhibit oxidative stress and DNA damage in Caco-2 monolayers of the coculture model.

### 3.4. Effect of Curcumin on Endoplasmic Reticulum Stress-Related Signaling Pathways

**Figure 4:**
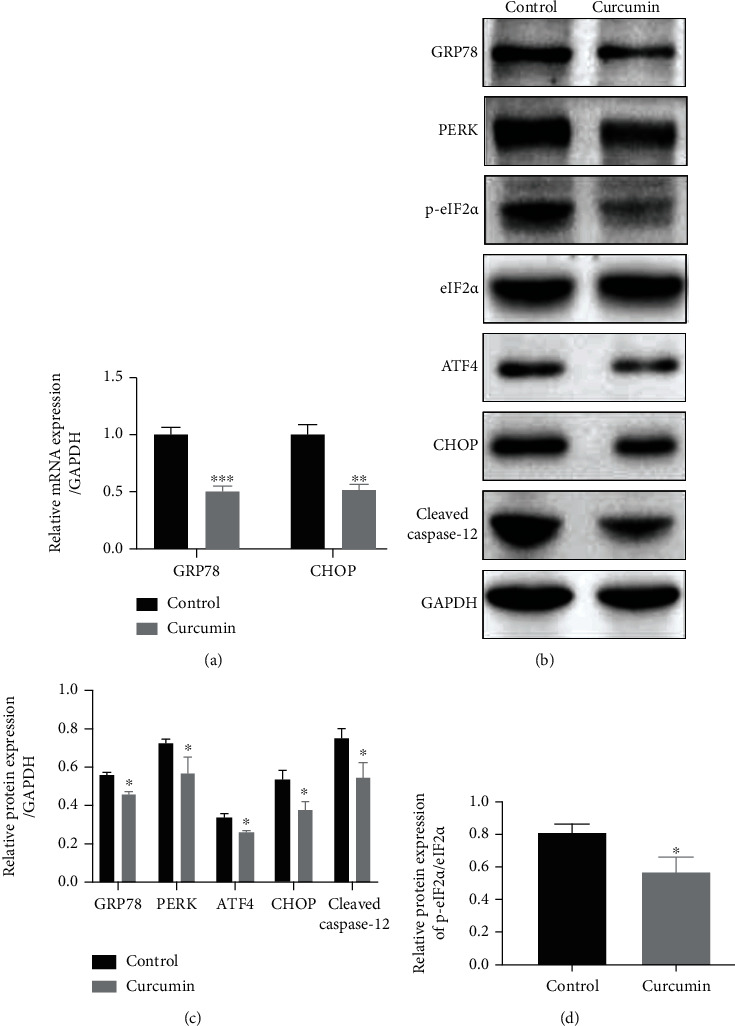
Curcumin treatment significantly inhibits the PERK-eIF2*α*-ATF4-CHOP signaling pathway and the cleavage of caspase-12. After curcumin treatment (10 *μ*M) for 48 h, (a) the mRNA levels of GRP78 and CHOP were detected by qRT-PCR, and (b) Western blot was performed to determine the protein levels of GRP78, PERK, p-eIF2*α*, eIF2*α*, ATF4, CHOP, and cleaved caspase-12. (c) Protein bands of GRP78, PERK, ATF4, CHOP, and cleaved caspase-12 were quantified by densitometry, and relative protein levels were normalized to GAPDH. (d) Protein bands of p-eIF2*α* were quantified by densitometry, and relative protein expression of p-eIF2*α* was normalized to eIF2*α*. Data are shown as the mean ± SD. ^∗^*P* < 0.05,  ^∗∗^*P* < 0.01, and^∗∗∗^*P* < 0.001. GRP78: glucose-regulated protein 78; CHOP: C/EBP homologous protein; PERK: protein kinase-like endoplasmic reticulum kinase; eIF2*α*: eukaryotic translation initiation factor 2*α*; ATF4: activating transcription factor 4; qRT-PCR: quantitative real-time polymerase chain reaction.

To explore the underlying mechanisms of the protective effects of curcumin on Caco-2 monolayers, we detected the expression of ERS-related molecules. GRP78 is an ER chaperone and a marker for ERS. The PERK-eIF2*α*-ATF4-CHOP signaling pathway and caspase-12 are closely related to ERS-induced apoptosis [10]. In the present study, we found that the mRNA expression levels of GRP78 and CHOP were significantly reduced after curcumin treatment (10 *μ*M) compared with those in the control group (Figure 4(a), *P* < 0.001 and *P* < 0.01). Western blot results showed that curcumin administration (10 *μ*M) significantly downregulated GRP78, PERK, ATF4, CHOP, and cleaved caspase-12 protein levels and inhibited eIF2*α* phosphorylation (Figures 4(b)–4(d), *P* < 0.05). Taken together, curcumin treatment significantly suppresses the PERK-eIF2*α*-ATF4-CHOP signaling pathway and the cleavage of caspase-12, thereby reducing apoptosis of IECs.

## 4. Discussion

In the present study, we found that curcumin could enhance epithelial barrier integrity of Caco-2 monolayers by upregulating TJ protein expressions and inhibiting apoptosis of IECs in an in vitro coculture model. The underlying mechanisms may be suppression of the PERK-eIF2*α*-ATF4-CHOP signaling pathway and the cleavage of caspase-12.

Curcumin is the major active component of turmeric, which belongs to the ginger family. This agent exerts various functions such as antioxidant, anti-inflammatory, antimicrobial, anticancer, antiarthritic, and antidiabetic activities [18]. It has been studied in a variety of human diseases including cardiovascular diseases, inflammatory diseases, metabolic diseases, and cancers [18]. Recently, several studies have reported the protective role of curcumin against experimental colitis [25, 26, 36]. Further, clinical studies showed that it was safe and effective in maintaining remission in patients with quiescent UC [28], and addition of curcumin to mesalamine therapy could significantly improve clinical and endoscopic remission in patients with active UC [29]. However, the underlying mechanisms of curcumin for the treatment of UC are not completely understood.

Recently, a few studies revealed that curcumin could improve the integrity of the intestinal epithelial barrier [37, 38]. The intestinal epithelium plays an essential role in maintaining intestinal homeostasis. Extensive studies have demonstrated that impairment of the intestinal barrier is a hallmark of UC and may contribute to the pathogenesis of UC [5–8]. The intestinal epithelial barrier consists of IECs and the paracellular apical junction complex, which includes TJs and adherence junctions [34]. Dysregulation of the TJs and destruction of IECs will lead to defects of intestinal epithelial barrier function and inflammation [39]. The TJ complex is mainly comprised of occludin, claudins, junctional adhesion molecules (JAMs), and ZO protein family. Decreased expression levels of ZO-1, occludin, and E-cadherin were detected in the intestinal epithelium of patients with UC [40]. Moreover, curcumin was reported to improve intestinal epithelial barrier function by enhancing ZO-1 and claudin-1 expressions in IECs and thereby reducing intercellular permeability [38]. Herein, our results showed that curcumin administration significantly upregulated the expression levels of ZO-1 and claudin-1 in Caco-2 monolayers (Figure 1), indicating that curcumin enhances intestinal epithelial barrier integrity by upregulating TJ protein expression.

Curcumin exhibits potent antioxidant properties. Excessive oxidative stress plays an important role in the pathogenesis of IBD [35]. iNOS, an isoform of nitric oxide synthase (NOS), is not expressed under normal physiological conditions. However, it can be induced and activated under pathological conditions, leading to an increase in nitric oxide (NO) production. NO can induce oxidative stress as it is recognized as a proinflammatory mediator and marker for oxidative stress status [41]. Previous studies reported that curcumin alleviated colitis in rats by reducing iNOS expression and NO production in the intestinal mucosa [42, 43]. It is well known that excessive oxidative stress leads to DNA double-strand breaks (DSBs), and *γ*H2AX is a marker for DNA DSB damage [44]. Immunohistochemistry analysis of colon specimens from IBD and colitis-associated cancer patients showed that *γ*H2AX-positive cells in inflamed and dysplastic mucosa were greatly enhanced compared to those in normal mucosa [45]. Moreover, the number of the cells was positively correlated with the degree of inflammation and grades of dysplasia, indicating that inflammation-driven colon carcinogenesis is related to oxidative DNA damage [45, 46]. Herein, we found that curcumin administration significantly reduced the expression levels of iNOS and *γ*H2AX in Caco-2 cells (Figure 3), indicating that curcumin exerts protective effects against oxidative stress and DNA damage in IECs in the coculture model.

The ER is the main organelle for the folding, modification, and export of secretory and transmembrane proteins and plays an important role in maintaining intracellular homeostasis [47]. Recent studies have indicated that ERS and the accompanying unfolded protein response (UPR) were commonly activated in the colon of patients with UC [13–15]. GRP78, also known as Bip, is an ER-resident chaperone and is regarded as a marker for ERS. Under stress-free conditions, GRP78 binds to three signal transduction factors (inositol-requiring enzyme 1 (IRE1), activated transcription factor 6 (ATF6), and PERK) and is maintained in its inactive state [10, 48]. Once ERS is detected, IRE1, ATF6, and PERK are released from GRP78 and become activated. Activated PERK phosphorylates eIF2*α* and reduces the activity of eIF2, thereby suppressing global translation of mRNAs and alleviating the burden on ER [10, 48]. eIF2a phosphorylation in IECs is required for maintaining normal intestinal barrier function [17]. Decreased phosphorylation of eIF2*α* was detected in the normal colonic mucosa of patients with UC [14]. Previous studies reported that curcumin could attenuate tissue damage and cell apoptosis induced by ERS by inhibiting PERK/eIF2*α*-mediated signaling pathways [22, 49]. Nevertheless, whether curcumin can improve intestinal barrier integrity in colitis by regulating ERS has not yet been reported. ATF4 is a main downstream target of eIF2*α* phosphorylation and regulates the expression of genes involved in protein folding, oxidative stress, and ERS-mediated apoptosis [45]. In the present study, we found that curcumin administration significantly downregulated the expression of ER chaperone GRP78 in Caco-2 cells. Moreover, the expression levels of PERK and ATF4 and the phosphorylation level of eIF2*α* were also remarkably reduced (Figure 4). Our findings suggest that curcumin may exert protective effects against excessive ERS in IECs by suppressing the PERK-eIF2*α*-ATF4 pathway.

Severe or prolonged ERS renders the UPR insufficient to restore ER homeostasis and lead to apoptosis. CHOP, a downstream target of the PERK-eIF2*α*-ATF4 pathway, is a key molecule in ERS-induced apoptosis. CHOP induces apoptosis by increasing proapoptotic protein expressions and decreasing antiapoptotic protein levels [10]. Enhanced expression of CHOP has been detected in the intestinal mucosa of patients with IBD [13]. IEC-specific overexpression of CHOP in mice impairs cell proliferation of IECs, leading to increased susceptibility toward dextran sodium sulfate- (DSS-) induced colitis [50]. Moreover, ablation of CHOP alleviated DSS-induced colitis in mice [51]. CHOP may aggravate colitis through macrophage infiltration, production of reactive oxygen species (ROS) and IL-1*β*, and aggravation of intestinal mucosal cell apoptosis. Caspase-12, a cysteine protease localized in the ER, is considered a mediator of ERS-induced apoptosis [10]. It can be activated by excessive ERS and subsequently activate downstream caspases including caspase-9 and caspase-3, ultimately resulting in apoptosis [10]. Previous studies indicated that curcumin could attenuate cell apoptosis by inhibiting ERS and downregulating GRP78 and CHOP protein expression [21, 22]. Herein, our results showed that curcumin significantly attenuated apoptosis of Caco-2 cell monolayers and downregulated the protein levels of CHOP and cleaved caspase-12 (Figures 2 and 4), suggesting that curcumin may reduce IEC apoptosis through inhibiting CHOP expression and caspase-12 cleavage.

It is worth mentioning that there are some limitations to our study. The major limitation is that we did not perform animal experiments; we only explored the effect and mechanisms of curcumin in an in vitro coculture model to mimic colitis. Our future work will focus on animal models of colitis such as trinitrobenzene sulfonic acid- (TNBS-) and DSS-induced colitis mice. Secondly, only a single concentration of curcumin was used in this study based on previous literature. Further exploration of the dose response of curcumin in the coculture cell model is required. Thirdly, our study only shows that curcumin could increase TJ protein production; however, intestinal epithelial barrier function was not evaluated. Additional functional experiments such as transendothelial electrical resistance (TEER) assay and fluorescein isothiocyanate-dextran permeability assay should be performed in the near future. Immunofluorescence and immunocytochemistry assays are also needed to evaluate the levels and locations of TJ protein expression in the future study. In addition, protein quantitation was determined by Western blot and ImageJ software in our study. Since Western blot is a semiquantitative technique, additional analyses to quantify protein expression levels are needed. Moreover, the exact molecular mechanisms of curcumin are not yet fully characterized; further experiments will be required in the future to clarify this issue.

## 5. Conclusion

In summary, curcumin enhanced intestinal epithelial barrier integrity by upregulating TJ protein expressions and inhibiting apoptosis of IECs via alleviation of ERS in an in vitro coculture cell model. Further, the PERK-eIF2*α*-ATF4-CHOP signaling pathway and the cleavage of caspase-12 were significantly suppressed upon curcumin treatment. Our findings showed a novel mechanism of curcumin for the treatment of colitis, which may provide evidence for the clinical application of curcumin in patients with UC.

## Figures and Tables

**Table 1:** Primer sequences for quantitative real-time polymerase chain reaction.

Gene name	Primer sequence
Homo GRP78 forward	5′-GGAACCACTCCCGTGGCATAA-3′
Homo GRP78 reverse	5′-CTTGGTAGGCACCACTGTGT-3′
Homo CHOP forward	5′-CACCACTCTTGACCCTGCTTCTC-3′
Homo CHOP reverse	5′-TGACCACTCTGTTTCCGTTTCC-3′
Homo GAPDH forward	5′-TGTTGCCATCAATGACCCCTT-3′
Homo GAPDH reverse	5′-CTCCACGACGTACTCAGCG-3′

GRP78: glucose-regulated protein 78; CHOP: C/EBP homologous protein; GAPDH: glyceraldehyde-3-phosphate dehydrogenase.

## Data Availability

The datasets generated during and/or analyzed during the current study are available from the corresponding author on reasonable request.
